# MiR-330-Mediated Regulation of SH3GL2 Expression Enhances Malignant Behaviors of Glioblastoma Stem Cells by Activating ERK and PI3K/AKT Signaling Pathways

**DOI:** 10.1371/journal.pone.0095060

**Published:** 2014-04-15

**Authors:** Yilong Yao, Yixue Xue, Jun Ma, Chao Shang, Ping Wang, Libo Liu, Wenjing Liu, Zhen Li, Shengtao Qu, Zhiqing Li, Yunhui Liu

**Affiliations:** 1 Department of Neurosurgery, Shengjing Hospital of China Medical University, Shenyang, Liaoning Province, People’s Republic of China; 2 Department of Neurobiology, College of Basic Medicine, China Medical University, Shenyang, Liaoning Province, People’s Republic of China; 3 Institute of Pathology and Pathophysiology, China Medical University, Shenyang, Liaoning Province, People’s Republic of China; 4 Department of Neurology, The First Affiliated Hospital, China Medical University, Shenyang, Liaoning Province, People’s Republic of China; H. Lee Moffitt Cancer Center & Research Institute, United States of America

## Abstract

MicroRNAs are currently considered as an active and rapidly evolving area for the treatment of tumors. In this study, we elucidated the biological significance of miR-330 in glioblastoma stem cells (GSCs) as well as the possible molecular mechanisms. SH3GL2 is mainly distributed in the central nervous system and considered to be a tumor suppressor in many tumors. In the present study, we identified miR-330 as a potential regulator of SH3GL2 and we found that it was to be inversely correlated with SH3GL2 expression in GSCs which were isolated from U87 cell lines. The expression of miR-330 enhanced cellular proliferation, promoted cell migration and invasion, and dampened cell apoptosis. When the GSCs were co-transfected with the plasmid containing short hairpin RNA directed against human *SH3GL2* gene and miR-330 mimic, we found that miR-330 promoted the malignant behavior of GSCs by down-regulating the expression of SH3GL2. Meanwhile, the ERK and PI3K/AKT signaling pathways were significantly activated, leading to the decreased expression of apoptotic protein and increased expression of anti-apoptotic protein. Furthermore, in orthotopic mouse xenografts, the mice given stable over-expressed SH3GL2 cells co-transfected with miR-330 knockdown plasmid had the smallest tumor sizes and longest survival. In conclusion, these results suggested that miR-330 negatively regulated the expression of SH3GL2 in GSCs, which promoted the oncogenic progression of GSCs through activating ERK and PI3K/AKT signaling pathways. The elucidation of these mechanisms will provide potential therapeutic approaches for human glioblastoma.

## Introduction

Glioblastoma is the most lethal type of primary brain tumor. Despite aggressive surgical treatment, followed by radiation and chemotherapy, the median survival time is less than 2 years [1]. Glioblastoma contains a small subpopulation of self-renewing tumorigenic cancer stem cells (glioblastoma stem cells, GSCs) that are implicated in tumor growth, recurrence, metastases as well as resistance to conventional therapies [2], [3], [4]. Understanding the mechanisms of these cells is essential for the development of therapeutic approaches for treatment of glioblastoma. In the present study, we mainly focused on the biological significance of GSCs isolated from U87 glioblastoma cell line.

MicroRNAs (miRNAs), a class of non-coding small molecule RNAs, participated in the regulation of individual development, apoptosis, proliferation and differentiation [5], [6]. Altered patterns of miRNA expression between normal and tumor tissues affected tumorous cell cycle and survival programs [7], [8], [9], [10]. MiR-330 was first discovered by Weber [11] and was reported to act as a tumor-suppressor in prostate and lung primary tumors [12], [13], [14]. However, the function and molecular mechanisms of miR-330 in the regulation of GSCs malignant behavior have still remained completely unknown.

SH3GL2 is mainly distributed in the central nervous system, particularly enriched in presynaptic ganglion whereas it may be expressed at very low expression levels in other tissues [15], [16]. It has been confirmed that SH3GL2 displayed a decreased expression level in many tumors such as non-small lung cancer and laryngeal carcinoma [17], [18]. Potter et al also reported the deletion of *SH3GL2* locus in pediatric pilocytic astrocytoma, suggesting that SH3GL2 might function as a tumor suppressor in brain tumors [19]. However, it’s still unclear why the expression level of SH3GL2 is decreased in cancer cells, especially in brain glioblastoma.

Soubeyran’s research showed that the complexes formed by SH3GL2, Cb1 and CIN85 activated the epidermal growth factor receptor (EGFR) on the cell membrane, mediated EGFR endocytosis and promoted its degradation [20]. About half of the glioblastomas exhibit EGFR anomalies including amplification and mutation of the *EGFR* gene and/or increased EGFR protein expression [21]. Downstream signaling pathways including mitogen-activated protein kinase (MAPK), phosphatidylinositol 3-kinase (PI3K/Akt), phospholipase C (PLC), signal transducer and activator transcription (STAT) and SRC/FAK pathways will be activated by ligand binding to the receptor [22], [23]. Decreased internalization and degradation of EGFR contributed to the enhanced proliferation, angiogenesis, necrosis, impaired apoptosis and treatment resistance [24], [25].

In this study, we mainly aimed to investigate the role of miR-330 in biological significance of GSCs and thereby to determine whether the ERK and PI3K/AKT pathways will be involved in miR-330-dependent regulation of malignant behavior of GSCs via down-regulating SH3GL2 expression.

## Materials and Methods

### Ethics Statement

This study was carried out in strict accordance with the recommendations in the Guide for the Care and Use of Laboratory Animals of the National Institutes of Health. The experimental protocol was approved by Administrative Panel on Laboratory Animal Care of the Shengjing Hospital. Mice were housed in an animal facility and were maintained in a temperature-controlled and light-controlled environment. All animals had access to food and water ad libitum. All surgery was performed under anesthesia, and all efforts were made to minimize suffering.

### Reagents and Cell Culture

Human glioblastoma cell lines U87 was obtained from Shanghai Institutes for Biological Sciences Cell Resource Center, grown in Dulbecco’s Modified Eagle Medium (DMEM) of high glucose with fetal bovine serum (FBS, Life Technologies Corporation, Paisley, UK). The cells attached and grew as a monolayer in flasks. When the cells reached the logarithmic growth phase, at a density of about 5–6×10^6^ live cells per flask, they were re-suspended in the serum-free medium consisted of DMEM/F12 (Life Technologies Corporation, Paisley, UK) supplemented with 20 ng/ml of basic fibroblast growth factor (bFGF, Life Technologies Corporation, Paisley, UK), 20 ng/ml of epidermal growth factor (EGF, Life Technologies Corporation, Paisley, UK) and B27 serum-free supplement (Life Technologies Corporation, Paisley, UK). Cells were then incubated in a 25 cm^2^ non-treated cell culturing flask. All cells were incubated in a 5% CO_2_ humidified incubator at 37°C.

### Isolation and Identification of GSCs

After cultured in the serum-free medium for 2 weeks, the primary spheres formed and reached 100–200 cells each, the cells remaining in the flasks were the non-glioblastoma stem cells (non-GSCs). The cultures were harvested by mechanical centrifugation, trypsinized into single-cell suspensions and plated into a 96-well plate for the subsphere-forming assay by limiting dilution as described previously [26], [27], [28]. Meanwhile, the monolayer cells remaining in the flasks after harvesting the spheres were transferred into medium without growth factors but permissive for differentiation. Spheres and differentiated cells were cultured in pre-coated chamber slides and fixed, and then were stained with the antibodies as follows: anti-Nestin (rabbit, monoclonal, IgG1, 1∶100, Santa Cruz Biotechnology, USA), anti-CD133 (mouse, monoclonal, IgG1, 1∶100, Santa Cruz Biotechnology, USA), anti-GFAP (rabbit, polyclonal, 1∶100, Abcam, MA, USA), anti-beta-tubulinIII (mouse, monoclonal, IgG1, 1∶100, Santa Cruz Biotechnology, USA). The primary antibodies were detected with cy3-conjugated anti-mouse and FITC-conjugated anti-rabbit IgG antibodies (1∶200, Beyotime Institute of Biotechnology, Jiangsu, China). Cells were also counterstained with DAPI to identify all nuclei.

### RNA Extraction and Real-time PCR

Total RNA was extracted using Trizol (Life Technologies Corporation, Carlsbad, CA, USA) according to the manufacturer’s instruction. To analyze miR-330 expression levels among different groups, the real-time PCR assay was used to quantify the miRNAs expression levels. Taqman MicroRNA Reverse Transcription Kit, Taqman Universal Master Mix II and TaqMan MicroRNA Assay of miR-330 and U6 (Applied Biosystems, Foster City, CA, USA) were used for reverse transcription and real-time PCR amplification. The expression levels of miR-330 were normalized with the reference U6, and fold changes were calculated by relative quantification (2^−ΔΔCt^) method.

### Transfection and Generation of Stable Cell Lines

Human full-length *SH3GL2* gene and its 3′ untranslated region (UTR) were constructed in pEX-2 plasmid vector (GenePharma, Shanghai, China). In order to knockdown *SH3GL2*, cells were transfected with the plasmid containing short hairpin RNA directed against human *SH3GL2* gene (sh*SH3GL2*) (GenePharma, Shanghai, China). The sequence of sh*SH3GL2* was Sence: 5′-CACCGCCTAGAAGGGAATATCAACCTTCAAGAGAGGTTGATATTCCCTTCTAGGCTTTTTTG-3′; Anti-sence: 5′-GATCCAAAAAAGCCTAGAAGGGAATATCAACCTCTCTTGAAGGTTGATATTCCCTTCTAGGC-3′. Stable transfection was performed at about 80% confluency in 24-well plates using Lipofectamine LTX and Plus Reagents (Life Technologies Corporation, Carlsbad, CA, USA) according to the manufacturer’s instructions. The stable transfected cells were selected by the culture medium containing 0.5 mg/ml G418 (Sigma-Aldrich, St Louis, MO, USA). After approximately 4 weeks, G418-resistant cell clones were established: SH3GL2 (+) and SH3GL2 (−).

### Cell Transfection of miRNA

The miRNA-330 mimics, miRNA-330 inhibitors and their negative control molecules were synthesized (Life Technologies Corporation, MD, USA). GSCs were transfected using lipofectamine 2000 reagent (Life Technologies Corporation, Carlsbad, CA, USA). Transfection complexes were prepared according to the manufacturer’s instructions and added directly to the cells to a final oligonucleotide concentration of 50 nM. After transfected 6 h, the medium was replaced with fresh serum-free medium of GSCs, and the cells were incubated for an additional 48 h. The transfected efficacy of miRNA-330 mimics and miRNA-330 inhibitors were evaluated by real-time PCR, and the highest transfected efficacy of these were both occurred at 48 h. Therefore, the time after transfected 72 h was considered as the harvested time in the subsequent experiments. GSCs transfected with miRNAs were divided into 5 groups: control group given no miRNAs, mock 1 group transfected with miR-330 mimics negative control molecules, pre-miR-330 group transfected with miR-330 mimics, mock 2 group transfected with miR-330 inhibitors negative control molecules, anti-miR-330 group transfected with miR-330 inhibitors. Those stable transfected cells co-transfected with miR-330 mimics (or miR-330 inhibitors) were divided into 5 groups: control group, SH3GL2 (−) & pre-miR-330 group (SH3GL2 (−) stable cells co-transfected with miR-330 mimics), SH3GL2 (−) & anti-miR-330 group (SH3GL2 (−) stable cells co-transfected with miR-330 inhibitors), SH3GL2 (+) & pre-miR-330 group (SH3GL2 (+) stable cells co-transfected with miR-330 mimics), SH3GL2 (+) & anti-miR-330 group (SH3GL2 (+) stable cells co-transfected with miR-330 inhibitors).

### Proliferation Assay

Cell proliferation was assessed using CCK8 (Beyotime Institute of Biotechnology, Jiangsu, China) according to the manufacturer’s instructions. Cells (2000 cells/well) were seeded into 96-well cell culture plates with five replicate wells for each group, and assayed at 72 h after transfection. 10% CCK8 was added into each well and incubated for another 4 h, and the absorbance was finally measured at the wavelength of 450 nm.

### Quantization of Apoptosis by Flow Cytometry

Apoptosis was determined using an Annexin V-fluorescein isothiocyanate assay (Beijing Baosai Biotech, China). The cells were washed twice in phosphate-buffered saline and re-suspended in a binding buffer. Annexin V-fluorescein isothiocyanate were added to the cell suspension, followed by incubation at room temperature in the dark for 15 min., and then 300 µl of binding buffer and 5 µl of propidium iodide (PI) were added. The cells were analyzed using flow cytometry (FACScan, BD Biosciences, USA). Data analysis was performed using CELLQuest 3.0 software.

### Cell Migration and Invasion Assay

24-well transwell chambers with 8 µm pore size polycarbonic membrane (Corning, NY, USA) were used to examine the effects of cell migration and invasion. Cells transfected with the desired miRNAs were dissociated enzymatically in 100µl serum-free medium and added to the upper chamber, while the lower chamber was filled with 600 µl medium containing 10% FBS. Cells on the top of membrane surface were removed with cotton swabs after incubation for 24 h at 37°C. Cells on the bottom of the membrane surface were fixed with methanol and glacial acetic acid in a 3∶1 ratio, then stained with 20% Giemsa solution for 30 min at room temperature and washed twice with PBS. Chambers were subjected to a microscopic inspection and counted within five randomly chosen microscope fields, and then the average number was taken. For the cell invasion ability assay, the procedure was similar to migration assay, but the top of membrane surface was coated with a 500 ng/µl Matrigel solution (BD, Franklin Lakes, NJ, USA), and incubated for 4 h at 37°C before added cells to the chamber for the further assay. The fix, stain and count of invasive cells were same as cell migration assay.

### Western Blot Analysis

Total protein was isolated in RIPA buffer supplemented with protease inhibitors and phosphatase inhibitor. The samples were centrifuged at 14,000×g 4°C for 5 min and the protein concentration of the supernatant extracts was determined by BCA protein assay kit (Beyotime Institute of Biotechnology, Jiangsu, China). Cell lysates were subjected to SDS-PAGE and proteins electroblotted onto a PVDF membrane. The membranes were blocked using 5% non-fat milk dissolved in Tris-buffered saline-Tween (TBST) containing 0.5% Tween 20 for 3 h. Thereafter, the membranes incubated with primary antibody as follows: SH3GL2 (rabbit, polyclonal, IgG1, 1∶500, Santa Cruz Biotechnology, CA, USA); ERK1/2, p-ERK1/2 (rabbit, monoclonal, IgG1, 1∶1000, Santa Cruz Biotechnology, CA, USA); PI3K, p-PI3K, AKT, p-AKT (rabbit, polyclonal, IgG1, 1∶1000, Cell Signaling Technology, Danvers, Massachusetts, USA), Caspase-3, TRAIL, XIAP, Bcl-2 (rabbit, polyclonal, IgG1, 1∶500, Santa Cruz Biotechnology, CA, USA) and GAPDH (mouse, monoclonal, IgG1, 1∶1000, Santa Cruz Biotechnology, USA). The membranes were then washed with TBST and incubated with correlated HRP-conjugated secondary antibody. Immunoblots were visualized by enhanced chemiluminescence (ECL kit, Santa Cruz Biotechnology, USA) and scanned using Chemi Imager 5500 V2.03 software. The relative integrated density values (IDVs) were calculated by Fluor Chen 2.0 software and normalized with GAPDH.

### Subcutaneous and Orthotopic Xenografts

For the in vivo xenograft assays, the stable transfected cell lines were used. The miR-330 knockdown plasmid, pEGFP-miR-330-inhibitor sponge (GenePharma, Shanghai, China), was transfected using Lipofectamine LTX and Plus Reagents and selected by the culture medium containing 10 µg/ml blasticidin (Life Technologies Corporation, Carlsbad, CA, USA) to generate miR-330 (−) stable transfected cell lines. To generate SH3GL2 (+) & miR-330 (−) stable transfected cell lines, pEGFP-miR-330-inhibitor sponge plasmids was transfected in SH3GL2 (+) stable transfected cells and selected by the culture medium containing 10 µg/ml blasticidin.

For subcutaneous implantation, mice were anesthetized by intra-peritoneal administration of ketamine plus xylazine. The mice were randomly divided into four groups: control group given only GSC cells, SH3GL2 (+) group given the SH3GL2 (+) stable transfected cells, miR-330 (−) group given the miR-330(−) stable transfected cells, SH3GL2 (+) & miR-330 (−) group given the SH3GL2 (+) & miR-330 (−) stable transfected cell lines. A total of 5×10^6^ cells suspended in 100µl 1×PBS was injected subcutaneously into the 4-week old BALB/C athymic nude mice (Cancer Institute of the Chinese Academy of Medical Science). Nude mice were monitored daily, and tumor size was measured with a caliper every five days, until 42 days post-injection. The tumor volume was calculated using the formula: tumor volumes (mm^3^) = (the longest diameter) × (the shortest diameter)^2^×0.5. Mice were sacrificed by CO_2_ inhalation and death was confirmed by cervical dislocation when tumors reached a volume of approximately 1200 mm^3^.

For orthotopic tumor inoculations, mice were anesthetized by intraperitoneal injection of ketamine plus xylazine. Mice were fixed in a stereotactic frame (Kopf Instruments, CA, USA) and a 1.5 cm (longitudinal) incision was made and a burr hole was drilled. 5×10^6^ cells suspended in 5µl 1×PBS were injected slowly into the right striatum using a gauge needle which remained in place for additional two minutes to minimize reflux through the needle track. The incision was closed with stitches. The mice were weighted, and their health and behavior was evaluated every two days. Mice were sacrificed by CO_2_ inhalation and death was confirmed by cervical dislocation if they exhibited excessive weight loss of 20% body weight, tumor metastasis, lethargy, or other signs of distress consisted with IACUC standards. In the 60-days observation period, the number of survived nude mice was recorded every 5 days.

### Statistical Analysis

Experimental data were expressed as means ± standard deviation (SD), and statistical analysis was performed using SPSS13.0 statistical software. One-way ANOVA followed by Dunnett’s post-test or *t*-test of two group comparisons was used for comparison between groups. *P*<0.05 was considered statistically significant.

## Results

### Isolation and Identification of GSCs from U87 Cells

**Figure 1:**
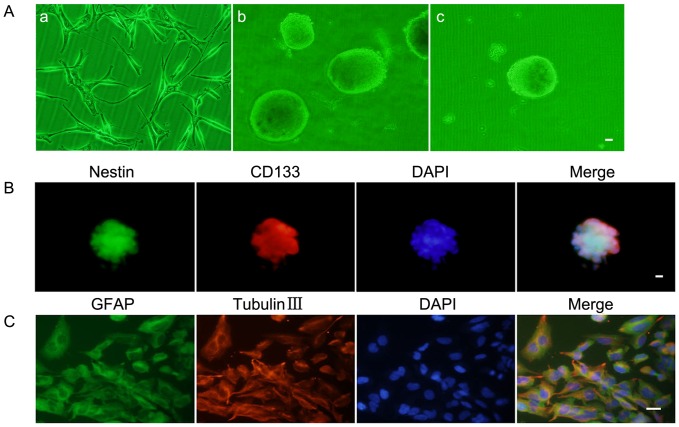
Isolation and identification of GSCs. (A) a: U87 cell lines attached and grew as a monolayer in flasks. b: The spheres formed and reached 100–200 cells each in the serum-free medium. c: Subsphere-forming assay of single-cell suspensions from the cell spheres. (B) Immunofluorescence staining of Nestin (green) and CD133 (red) in cell spheres. (C) Glioblastoma sphere-differentiated progenies were stained with GFAP (green) and beta-tubulin III (red). Scale bars represent 20 µm.

The U87 cells were expanded in the cultural medium containing FBS (Figure 1A–a). After cultured in the serum-free medium for 2 weeks, a lot of suspended cell masses appeared and gradually formed spheres containing 100–200 cells (Figure 1A–b). In order to test the self-renewing ability, the subspheres were harvested and a second round subsphere-forming assay was performed. The majority of cells formed spheres again after 1 week which was similar to those of the previous assay (Figure 1A–c). The stain of Nestin and CD133 proved that most cells within spheres expressed neural stem cell lineage markers on membranes (Figure 1B). Differentiation capacity was checked by differentiation assay and results showed that single-cell suspensions of spheres differentiated towards neuronal and astrocytic lineages and were positive for GFAP and beta-tubulin-III lineage markers (Figure 1C).

### The Expression of miR-330 was Increased in GSCs

**Figure 2:**
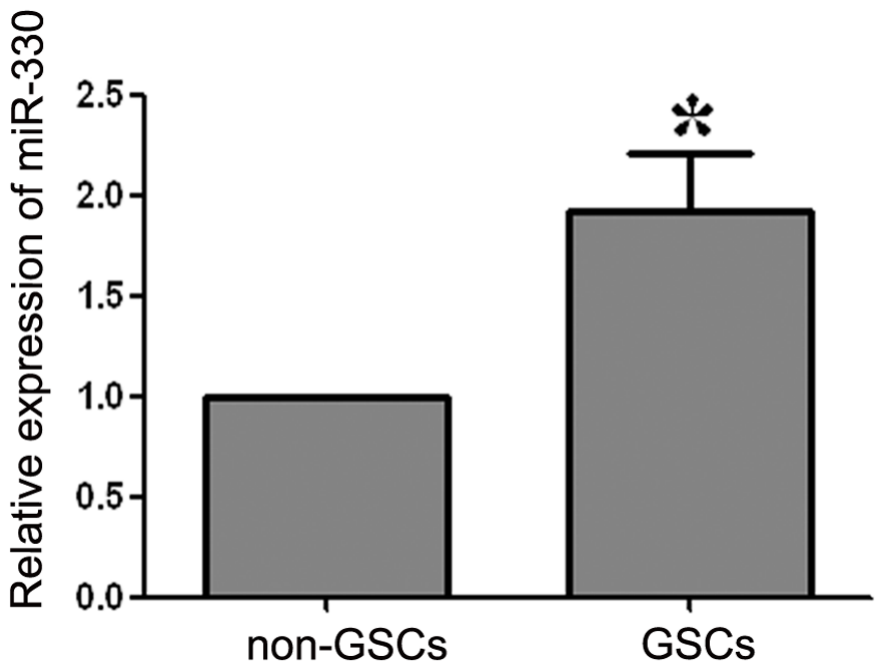
Expression of miR-330 in GSCs and non-GSCs. Relative expression levels of miR-330 in GSCs and non-GSCs. The expression of miR-330 in non-GSCs accounted for 45.26% of that in GSCs. Values represent the mean ± SD from five independent experiments. *P<0.05 vs. non-GSCs group.

Results of real-time PCR analysis showed that the miR-330 expression level was significantly higher in GSCs than non-GSCs (Figure 2).

### MiR-330 Promoted Proliferation, Anti-apoptosis, Migration and Invasion of GSCs

**Figure 3:**
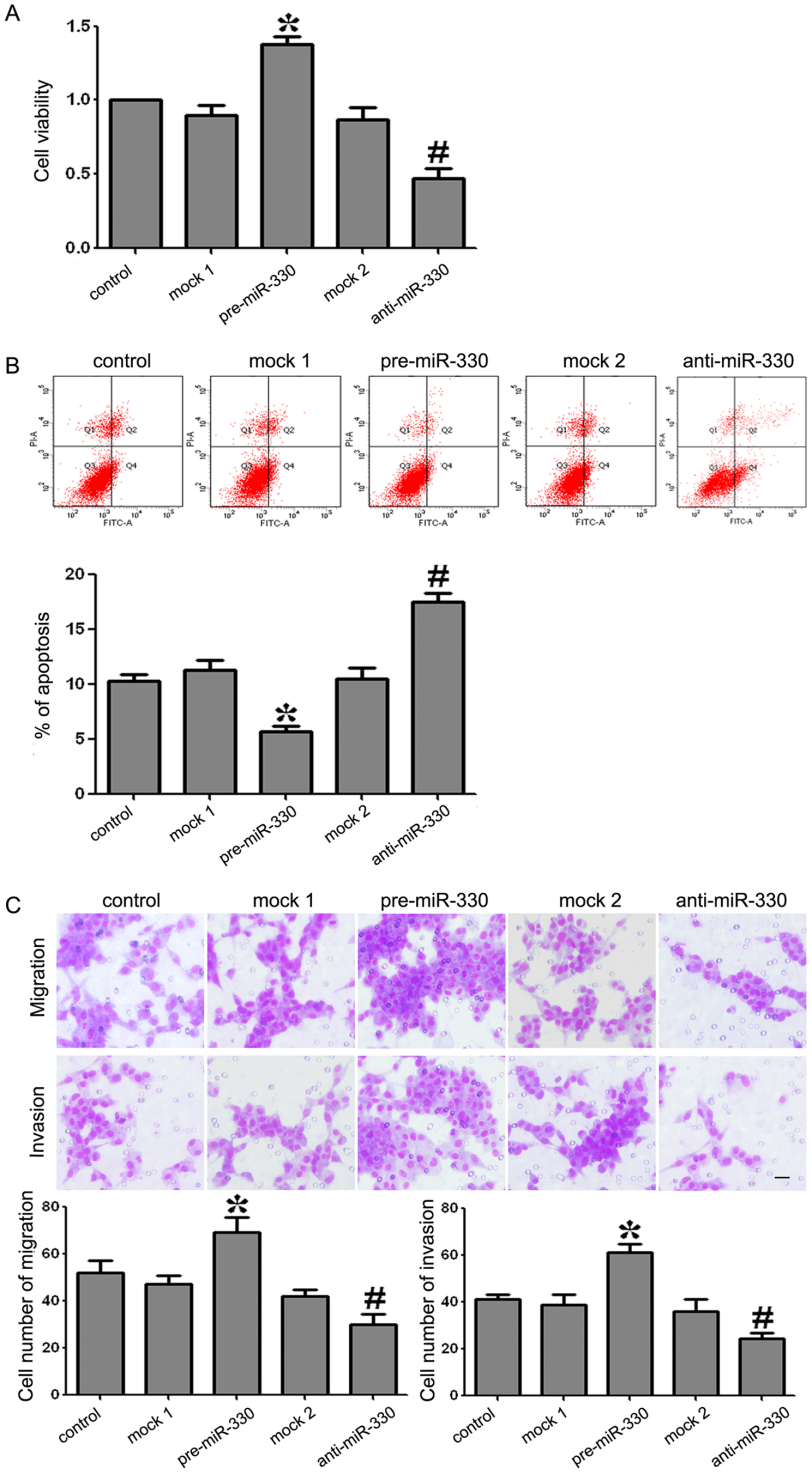
Proliferation, apoptosis, migration and invasion of GSCs transfected with miR-330 mimics (or miR-330 inhibitors). (A) CCK8 assay showed that the ability of GSCs proliferation with the expression of miR-330 changed. (B) Incidence of apoptotic cells was studied by flow ctometry. The cells were stained with annexin V-fluorescein isothiocyanate and counterstained with PI. (C) The ability of GSCs migration and invasion with the expression of miR-330 changed. The photographs about cells on the membrane and accompanying statistical plots were presented. Values represent the mean ± SD from five independent experiments. *P<0.05 vs. mock 1 group, *^#^P<*0.05 vs. mock 2 group.

To test the effects of miR-330 on GSCs proliferation, CCK8 assays were performed. As shown in Figure 3A, the proliferative ability of GSCs was increased significantly in pre-miR-330 group compared with the mock 1 group (*P<*0.05). There was no significant difference between the control group and the mock1 group. On the contrary, the proliferation of GSCs in anti-miR-330 group was decreased significantly compared with the mock 2 group (*P<*0.05). There was no significant difference between the control group and the mock 2 group.

To determine whether miR-330 was associated with GSCs apoptosis, quantization of apoptosis was assessed using flow cytometry. As shown in Figure 3B, the apoptosis rates in mock 1 and pre-miR-330 groups were 10.3±1.32% and 5.2±0.97% respectively. The apoptosis rate was significantly decreased by 5.1% in pre-miR-330 group compared with mock 1 group (*P<*0.05). There was no significant difference between the control group and the mock 1 group. The apoptosis rates in mock 2 and anti-miR-330 groups were 11.2±1.58% and 17.2±2.04% respectively. The apoptosis rate was significantly increased by 6% in anti-miR-330 group compared with the mock 2 group (*P<*0.05). There was no significant difference between the control group and the mock 2 group.

Transwell assays were preformed to test migration and invasion abilities of GSCs. As shown in Figure 3C, the migration and invasion were promoted in the pre-miR-330 group compared with the mock 1 group (*P*<0.05). There was no significant difference between the control group and the mock1 group. However, the migration and invasion were decreased in anti-miR-330 group compared with the mock 2 group (*P*<0.05). And there was no significant difference between the control group and the mock 2 group. These results clearly revealed that miR-330 could enhance FBS-induced migration and invasion of GSCs.

### The Over-expressed MiR-330 Inhibited the Expression of SH3GL2 in GSCs

**Figure 4:**
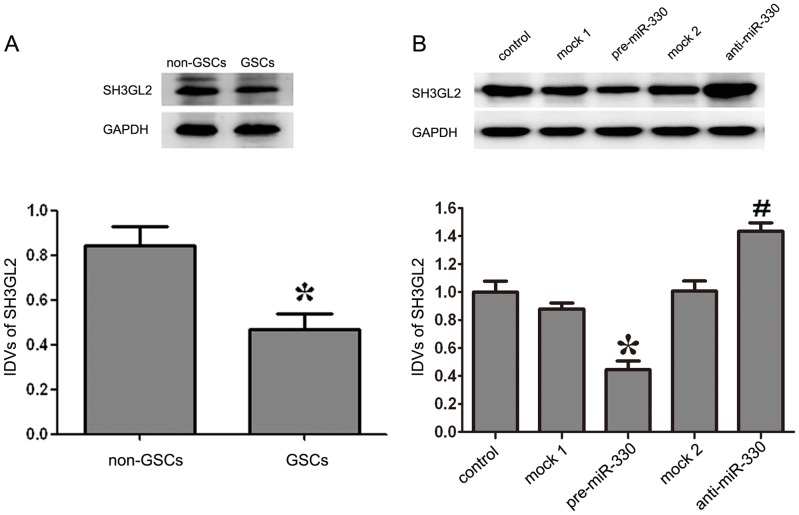
Expression of SH3GL2 in GSCs and non-GSCs, and SH3GL2 expression with expression of miR-330 changed. (A) The expression levels of SH3GL2 in GSCs and non-GSCs, using GAPDH as an endogenous control. The IDVs of SH3GL2 protein levels are shown. Data represent means ± SD (n = 5, each). *P<0.05 vs. non-GSCs group. (B) Western blot analysis for miR-330 regulated the expression of SH3GL2 in GSCs, using GAPDH as an endogenous control. The IDVs of SH3GL2 protein levels are shown. Values represent the mean ± SD from five independent experiments. *P<0.05 vs. mock 1 group, *^#^P<*0.05 vs. mock 2 group.

As shown in Figure 4A, the expression level of SH3GL2 was lower in GSCs than in non-GSCs. The expression levels of SH3GL2 were detected by Western blot. As shown in Figure 4B, the protein expression levels of SH3GL2 were decreased in the pre-miR-330 group compared with the mock 1 group (*P<*0.05). There was no obvious difference between the control group and the mock 1 group. By contrast, the anti-miR-330 group displayed the opposite effect on SH3GL2 expression to pre-miR-330 group: the expression level of SH3GL2 was increased compared with that of the mock 2 group (*P<*0.05). Similarly, there was no obvious difference between the control group and the mock 2 group. All the results indicated that the expression of miR-330 could inhibit SH3GL2 expression at protein level.

### MiR-330 Promoted Proliferation, Anti-apoptosis, Migration and Invasion of GSCs by Down-regulating SH3GL2

**Figure 5:**
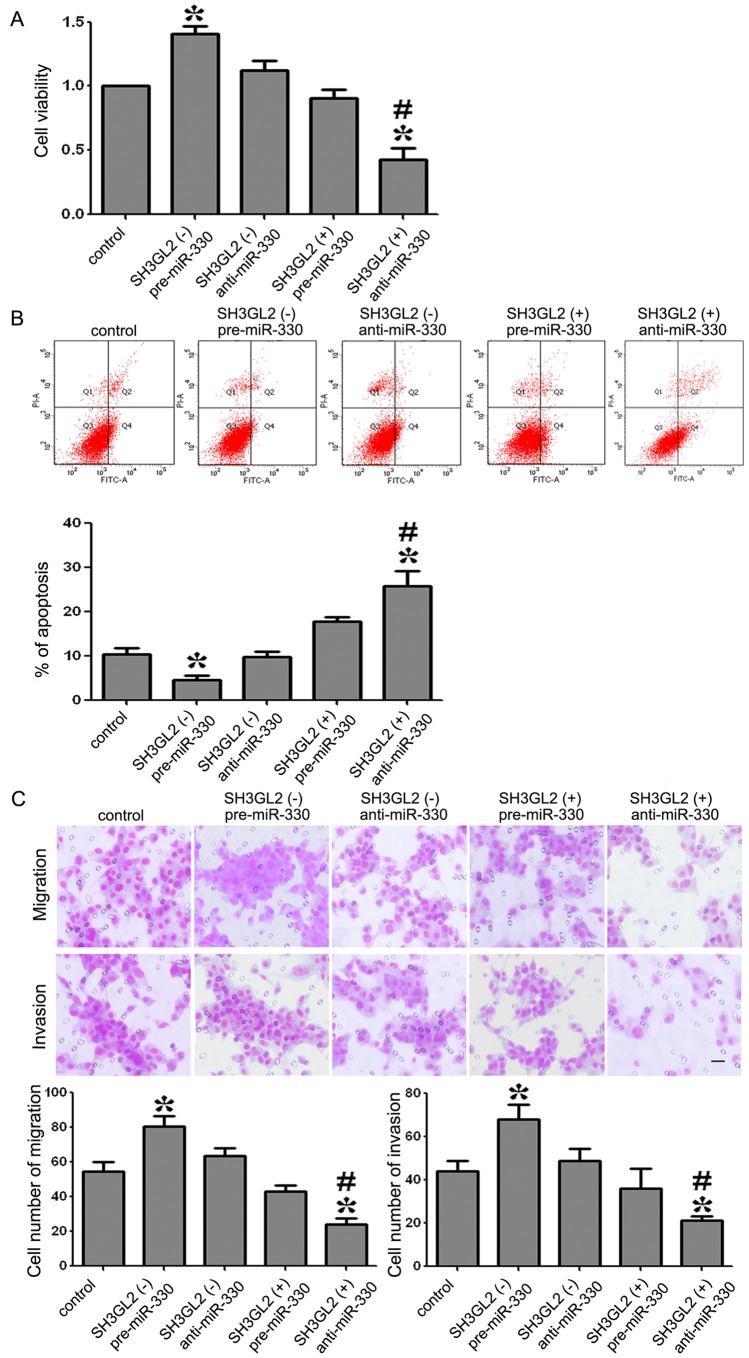
Proliferation, apoptosis, migration and invasion of GSCs with the expression of miR-330 and SH3GL2 changed. (A) The ability proliferation in GSCs with the expression of miR-330 and SH3GL2 changed. (B) Incidence of apoptotic cells was studied by flow ctometry. The cells were stained with annexin V-fluorescein isothiocyanate and counterstained with PI. (C) The ability of GSCs migration and invasion with the expression of miR-330 and SH3GL2 changed. Values represent the mean ± SD from five independent experiments. *P<0.05 vs. control group, *^#^P<*0.01 vs. *SH3GL2* (−) & pre-miR-330 group. Scale bars represent 20 µm.

As shown in Figure 5A, the cellular viability of the SH3GL2 (−) & pre-miR-330 group was increased whereas that of SH3GL2 (+) & anti-miR-330 group was decreased compared with the control group. Furthermore, the cellular viability was obviously lower in SH3GL2 (+) & anti-miR-330 group than SH3GL2 (−) & pre-miR-330 group (*P<*0.05). These results demonstrated that miR-330 promoted GSCs proliferation by down-regulating SH3GL2.

As shown in Figure 5B, the apoptosis rates in control and SH3GL2 (−) & pre-miR-330 groups were 11.9±1.92% and 3.2±0.39% respectively, which means that the apoptosis rate was significantly decreased by 8.7% in SH3GL2 (−) & pre-miR-330 group compared with the control group. The apoptosis rate in SH3GL2 (+) & anti-miR-330 was 24.1±3.87%, which was significantly increased by 12.2% and 20.9% compared with the control or SH3GL2 (−) & pre-miR-330 groups. These results demonstrated that miR-330 inhibited apoptosis of GSCs by down-regulating SH3GL2.

As shown in Figure 5C, the migration and invasion of GSCs was promoted in SH3GL2 (−) & pre-miR-330 group compared with the control group whereas that of the SH3GL2 (+) & anti-miR-330 group was decreased. In the SH3GL2 (+) & anti-miR-330 group, the migration and invasion of GSCs was decreased compared with the SH3GL2 (−) & pre-miR-330 group (*P*<0.05). These results strongly suggested that miR-330 was an oncogenic factor that was involved in the migration and invasion of GSCs via down-regulating SH3GL2 expression.

### MiR-330 Induced the Activation of ERK and PI3K/AKT Pathways by Down-regulating SH3GL2

**Figure 6:**
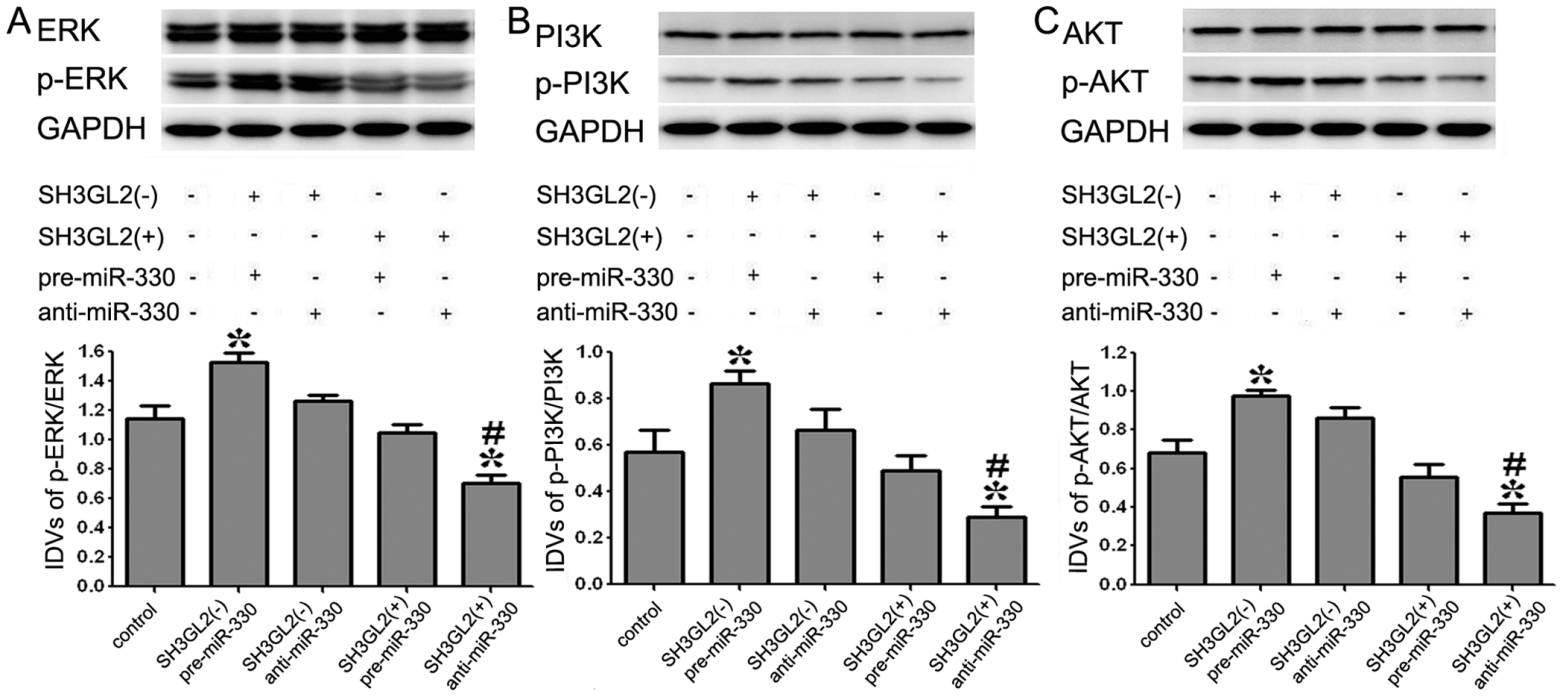
Expression of p-ERK/ERK and PI3K/AKT in GSCs with the expression of miR-330 and SH3GL2 changed. (A) The expression levels of p-ERK/ERK with the expression of miR-330 and SH3GL2 changed. (B, C) The expression levels of PI3K/AKT pathway within the expression of miR-330 and SH3GL2 changed. GAPDH was used as an internal loading control. Accompanying graphs show densitometry analysis of protein expression. Values represent the mean ± SD from five independent experiments. *P<0.05 vs. control group, *^#^P<*0.01 vs. *SH3GL2* (−) & pre-miR-330 group.

To test whether the ERK and PI3K/AKT pathways were activated in this process, Western blot assay was performed. The results showed that there was an increase of p-ERK/ERK expression in SH3GL2 (−) & pre-miR-330 group compared with the control group, and a decrease in SH3GL2 (+) & anti-miR-330 group. The expression of p-ERK/ERK was decreased in SH3GL2 (+) & anti-miR-330 group compared with SH3GL2 (−) & pre-miR-330 group (*P<*0.05) (Figure 6A). The analysis of PI3K/AKT pathway also confirmed similar changes in GSCs (Figure 6B, C). These results demonstrated that the mechanism of miR-330 induced malignant behavior of GSCs was associated with the activation of ERK and PI3K/AKT pathways via down-regulating SH3GL2.

### MiR-330 Inhibited the GSCs Apoptosis by Regulating the Expression of Apoptotic Proteins through Down-regulating SH3GL2

**Figure 7:**
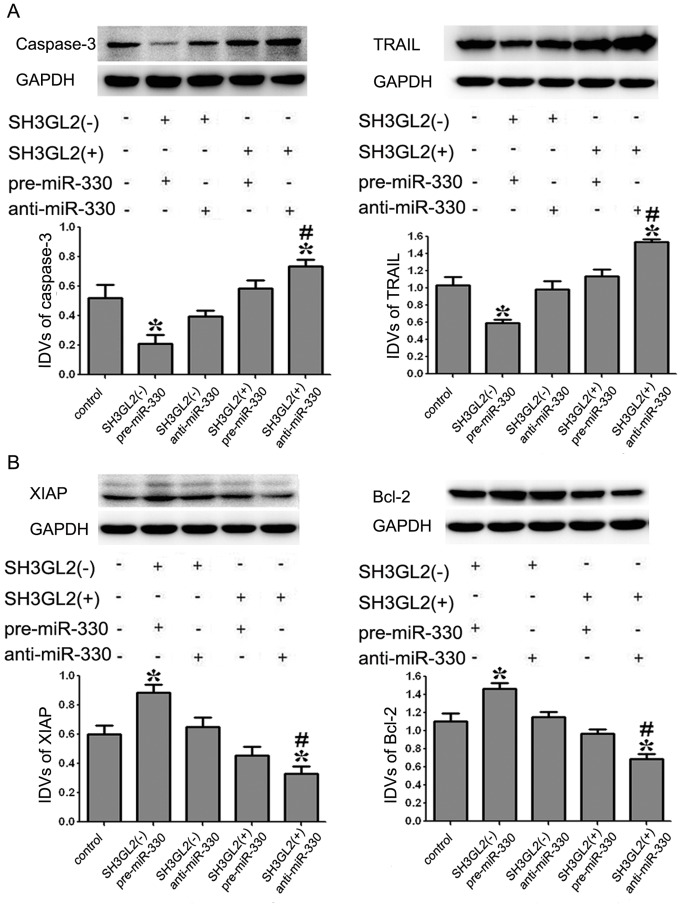
Expression levels of apoptotic proteins in GSCs with the expression of miR-330 and SH3GL2 changed. (A) The expression levels of caspase-3 and TRAIL in GSCs with the expression of miR-330 and SH3GL2 changed. (B) The expression levels of XIAP and Bcl-2 in GSCs with the expression of miR-330 and SH3GL2 changed. Accompanying graphs show densitometry analysis of protein expression. Values represent the mean ± SD from five independent experiments. *P<0.05 vs. control group, *^#^P<*0.01 vs. *SH3GL2* (−) & pre-miR-330 group.

To determine whether apoptotic proteins were involved in the GSCs apoptosis induced by miR-330 via the down-regulation of SH3GL2, the protein expression levels of apoptotic proteins were measured by Western blot where GAPDH was used as an internal loading control. As shown in Figure 7A, the results demonstrated that the protein expression levels of Caspase-3 and TRAIL were inhibited in SH3GL2 (−) & pre-miR-330 group compared with the control group (*P<*0.05). However, the expression of them in SH3GL2 (+) & anti-miR-330 group showed an increase compared with the control group (*P<*0.05). The protein expression levels of Caspase-3 and TRAIL in SH3GL2 (+) & anti-miR-330 group were significantly promoted compared with SH3GL2 (−) & pre-miR-330 group (*P<*0.05). As shown in Figure 7B, the analysis of anti-apoptotic protein showed that the protein expression levels of XIAP and Bcl-2 were promoted in SH3GL2 (−) & pre-miR-330 group compared with the control group (*P<*0.05). However, the expression of them in SH3GL2 (+) & anti-miR-330 group showed a decrease compared with the control group (*P<*0.05). Meanwhile, Bcl-2 and XIAP expression was inhibited in SH3GL2 (+) & anti-miR-330 group compared with SH3GL2 (−) & pre-miR-330 group (*P<*0.05). These data confirmed that miR-330 played an anti-apoptotic role in GSCs by down-regulating SH3GL2.

### Knockdown MiR-330 and Over-expressed SH3GL2 Suppressed Tumor Growth and Prolonged Survival in vivo

**Figure 8:**
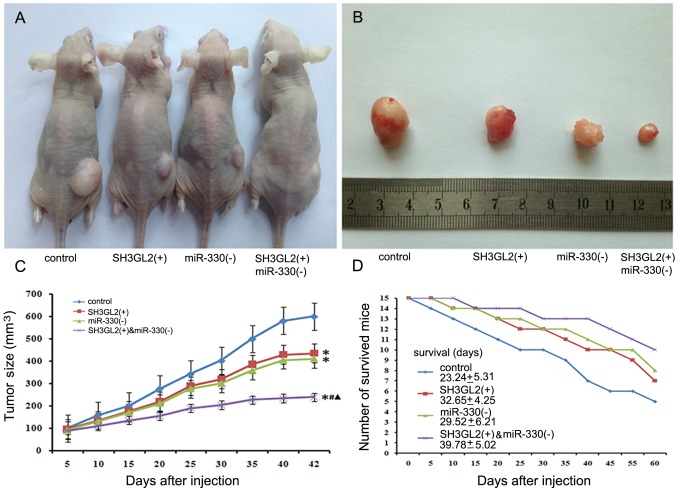
Tumorigenicity assay in nude mice. (A) Nude mice were subcutaneously injected in the right flank with control cells, SH3GL2 (+) stable transfected cells, miR-330 (−) stable transfected cells and SH3GL2 (+) & anti-miR-330 stable transfected cells. (B) A sample tumor from representative group at 42 days post-injection was shown. (C) Tumor growth curve in nude mice. After subcutaneous implantation, tumor volume was measured every 5 days in mm^3^ (*n = *5). *P<0.05 vs. control group, *^#^P<*0.05 vs. SH3GL2 (+) group, *^▴^P<*0.05 vs. miR-330 (−) group. (D) Numbers of survived mice for 60 days (*n = *15).

As shown in Figure 8A, B, C, the results showed that the tumor sizes were smaller in the SH3GL2 (+) and miR-330 (−) groups compared with the control group. The smallest tumor sizes were observed in SH3GL2 (+) & miR-330 (−) group (*P*<0.05). There was no significant difference between the SH3GL2 (+) group and the miR-330 (−) group. Compared with the SH3GL2 (+) group or miR-330 (−) group, mice in SH3GL2 (+) & miR-330 (−) group had longer survival (Figure 8D).

## Discussion

In this study, we proved that the expression level of miR-330 was increased in GSCs compared with non-GSCs. Besides, miR-330 could promote the ability of proliferation, anti-apoptosis, migration and invasion in GSCs. However, the expression of SH3GL2 was lower in GSCs, and could be inhibited by miR-330. In order to investigate whether SH3GL2 was involved in the miR-330-induced regulation of the malignant behavior of GSCs, the expression level of miR-330 and SH3GL2 were altered. Data demonstrated that SH3GL2 was negatively regulated by miR-330 in GSCs, which promoted the malignant behavior of GSCs. Further, we found that the ERK and PI3K/AKT signaling pathways were activated leading to regulate the expression of both apoptotic and anti-apoptotic proteins effectively blocking the apoptotic pathway. The in vivo studies also showed that mice carrying knockdown miR-330 and over-expressed SH3GL2 tumors produced significantly smallest tumors and had a highest survival.

In the present study, we have found that miR-330 had a higher expression level in GSCs, which indicated that it played an oncogenic role in the GSCs. In order to investigate the effects of miR-330 on GSCs malignant behavior, we carried out proliferation assay, flow cytometry, cell migration and invasion assays. The results showed that GSCs treated with pre-miR-330 promoted the abilities of proliferation, anti-apoptosis, migration and invasion. Conversely, the GSCs treated with anti-miR-330 had lower abilities of malignant behavior mentioned above. Recently, our preliminary research also confirmed similar results in human glioblastoma tissues and cells [29]. However, other studies described it as a tumor-suppressor in prostate cancer and colorectal carcinoma [12], [30], [31]. The reason for this was that certain microRNA may be oncogenic in one cell or tissue type but tumour-suppressive in another, depending on the tissue context and target genes [32].

Our results proved that SH3GL2 had a reduced expression level in the GSCs compared with non-GSCs, suggesting that SH3GL2 was a tumor suppressor in GSCs. Consistent with this possibility, analyses in head and neck squamous cell carcinoma, breast carcinoma, laryngeal carcinoma and urothelial carcinoma have implicated SH3GL2 as a candidate tumor suppressor [18], [33], [34], [35]. Santanu also reported that genomic loss of *SH3GL2* and its expression appeared to be an important and common inactivation event in lung cancer progression [17]. To elucidate the regulation of miR-330 on SH3GL2 expression, GSCs were transfected with miR-330 mimics or miR-330 inhibitors. The protein expression levels of SH3GL2 in pre-miR-330 group were decreased while that it in anti-miR-330 group were increased. The results indicated that miR-330 could inhibit SH3GL2 expression at protein level. MiRNAs can regulate gene expression by inducing direct cleavage of the targeted mRNAs or inhibiting translation through perfect or nearly perfect complementarity to targeted mRNAs at the 3′-UTRs in animals [36], [37]. Published article from our lab has shown that miR-330 could directly bind to the 3′-UTR of *SH3GL2* [29].

Nest, we aimed to confirm whether SH3GL2 was also involved in the regulation of malignant behavior of GSCs. Result showed that GSCs treated with SH3GL2 (+) & anti-miR-330 showed lower abilities of proliferation, anti-apoptosis, migration and invasion than GSCs treated with SH3GL2 (−) & pre-miR-330. Conversely, GSCs treated with SH3GL2 (−) & pre-miR-330 had higher abilities of these. This result strongly indicated that over-expression of miR-330 could increase the malignant behavior of GSCs via the down-regulating SH3GL2. The in vivo studies also confirmed that mice carrying knockdown miR-330 and over-expressed SH3GL2 tumors produced significantly smallest tumors and had a highest survival. Studies have already shown that SH3GL2, also known as endophilin-1, might activate the EGFR on the cell membrane, enhance the endocytosis of the EGFR and trigger its degradation by forming CIN85-SH3GL2-Cb1 complex [20]. Low expression of SH3GL2 may delay the degradation of EGFR, thereby enhancing the functions of EGF. The binding of EGF to EGFR could activate MAPK pathway which can regulate fundamental cellular processes such as growth, proliferation, differentiation, migration and apoptosis. [38], [39], [40]. MAPKs are a superfamily of protein serine–threonine kinases including ERKs. In addition, previous study found that EGF-induced receptor phosphorylation triggered the activation of downstream PI3K/Akt pathway which resulted in the change of various biological effects [41], [42]. To further clarify the potential mechanisms in the SH3GL2-dependent regulation of malignant behavior of GSCs, the detection of ERK and PI3K/AKT activity was carried out by Western blot after the expression of miR-330 and SH3GL2 were changed. Results showed that the highest activity of ERK and PI3K/AKT appeared in SH3GL2 (−) & pre-miR-330 group. On the contrary, the lowest activity of ERK and PI3K/AKT appeared in SH3GL2 (+) & anti-miR-330 group. The results proved that ERK and PI3K/AKT signaling pathways were involved in the oncogenic progression of GSCs since miR-330 negatively regulated the expression of SH3GL2. Ramjaun et al reported that SH3GL2 played a functional role in germinal center kinase-like kinase (rGLK)-mediated c-Jun N-terminal kinase (JNK) activation, suggesting its additional or perhaps complementary role in the JNK-dependent signaling pathway, which is widely involved in the regulation of cell proliferation, apoptosis and metabolic process [43], [44]. Additionally, recent studies have shown that SH3GL2 blocks the EGFR signaling and is associated with CTNNB1-mediated trans-activation pathway by reciprocally interacting with USP9X, a deubiquitinase stabilizing β-catenin, resulting in decreased tumor growth and invasion [17], [45]. These phenomena indicated that JNK and WNT signaling pathway might be also involved in oncogenic progression of GSCs since miR-330 negatively regulated the expression of SH3GL2.

Study had shown that the increased gene transcription caused by the activation of MAPK pathway leads to the increased expression levels of the anti-apoptotic protein Bcl-2 and apoptosis inhibitor protein XIAP [46]. EGF binding to its receptor leads to the activation of AKT and phosphorylation of AKT substrates including the Bcl-2 family member BAD. This blocks TRAIL-induced apoptosis by inhibiting the release of cytochrome c from the mitochondria and caspase-3-like activation [47]. In addition, EGF-induced activation of the PI3K signaling pathway is required for the prevention of TRAIL-induced apoptosis [48], [49]. TRAIL-mediated apoptosis can result in the activation of caspase-8 and -10, which induces apoptosis by subsequent activation of the executioner caspase-3 [50]. XIAP can prevent apoptosis by binding to it and inhibition of activated caspase-3 [51], [52]. Further, we investigated whether the changed expression levels of miR-330 and SH3GL2 might affect the expression of apoptotic proteins in GSCs. Our results showed that the SH3GL2 (−) & pre-miR-330 group showed a decreased expression of apoptotic protein Caspase-3 and TRAIL, and an increased expression of anti-apoptotic protein XIAP and Bcl-2. On the contrary, SH3GL2 (+) & anti-miR-330 group showed an increased expression of apoptotic protein Caspase-3 and TRAIL, and a decreased expression of anti-apoptotic protein XIAP and Bcl-2. This indicated that miR-330 could promote the malignant behavior of GSCs and regulate the expression of apoptotic protein through down-regulating the expression of SH3GL2 and activating ERK and PI3K/AKT signaling pathways. In addition to the co-regulation of apoptotic proteins mentioned above, PI3K can also trigger ERK signaling pathways by activating RAS [53]. These signal pathways are regulated by various positive and negative crosstalk and feedback loops [54], [55]. It was illustrated that there might be interactions between these signaling pathways in GSCs, which needs to be further investigated.

## Conclusion

This study proved for the first time that the tumor suppressor SH3GL2 is negatively regulated by miR-330 in GSCs. Over-expressed miR-330 could promote cell proliferation, anti-apoptosis, migration and invasion of GSCs through down-regulating SH3GL2 expression. The activation of the ERK and PI3K/AKT pathways as well as the changes in the expression of apoptotic proteins were the potential mechanisms involved in this process. The elucidation of these mechanisms will provided potential therapeutic approaches for human glioblastoma.

## References

[1] Stupp R, Mason WP, van den Bent MJ, Weller M, Fisher B, et al (2005) Radiotherapy plus concomitant and adjuvant temozolomide for glioblastoma. N Engl J Med 352: 987–996. 15758009 10.1056/NEJMoa043330

[2] Ji J, Black KL, Yu JS (2010) Glioma stem cell research for the development of immunotherapy. Neurosurg Clin N Am 21: 159–166. 19944974 10.1016/j.nec.2009.08.006 PMC2786895

[3] Fukaya R, Ohta S, Yamaguchi M, Fujii H, Kawakami Y, et al (2010) Isolation of cancer stem-like cells from a side population of a human glioblastoma cell line, SK-MG-1. Cancer Lett 291: 150–157. 19913993 10.1016/j.canlet.2009.10.010

[4] Pollard SM, Yoshikawa K, Clarke ID, Danovi D, Stricker S, et al (2009) Glioma stem cell lines expanded in adherent culture have tumor-specific phenotypes and are suitable for chemical and genetic screens. Cell Stem Cell 4: 568–580. 19497285 10.1016/j.stem.2009.03.014

[5] Garzon R, Calin GA, Croce CM (2009) MicroRNAs in Cancer. Annu Rev Med 60: 167–179. 19630570 10.1146/annurev.med.59.053006.104707

[6] Nimmo RA, Slack FJ (2009) An elegant miRror: microRNAs in stem cells, developmental timing and cancer. Chromosoma 118: 405–418. 19340450 10.1007/s00412-009-0210-z PMC4322900

[7] Yu X, Song H, Xia T, Han S, Xiao B, et al (2013) Growth inhibitory effects of three miR-129 family members on gastric cancer. Gene. 10.1016/j.gene.2013.09.048 24055727

[8] Callegari E, Elamin BK, Sabbioni S, Gramantieri L, Negrini M (2013) Role of microRNAs in hepatocellular carcinoma: a clinical perspective. Onco Targets Ther 6: 1167–1178. 24039437 10.2147/OTT.S36161 PMC3770717

[9] Koshkin PA, Chistiakov DA, Chekhonin VP (2013) Role of microRNAs in mechanisms of glioblastoma resistance to radio- and chemotherapy. Biochemistry (Mosc) 78: 325–334. 23590435 10.1134/S0006297913040019

[10] Palumbo S, Miracco C, Pirtoli L, Comincini S, (2013) Emerging roles of microRNA in modulating cell-death processes in malignant glioma. J Cell Physiol. 10.1002/jcp.24446 23929496

[11] Weber MJ (2005) New human and mouse microRNA genes found by homology search. FEBS J 272: 59–73. 15634332 10.1111/j.1432-1033.2004.04389.x

[12] Lee KH, Chen YL, Yeh SD, Hsiao M, Lin JT, et al (2009) MicroRNA-330 acts as tumor suppressor and induces apoptosis of prostate cancer cells through E2F1-mediated suppression of Akt phosphorylation. Oncogene 28: 3360–3370. 19597470 10.1038/onc.2009.192

[13] Pang Y, Young CY, Yuan H (2010) MicroRNAs and prostate cancer. Acta Biochim Biophys Sin (Shanghai) 42: 363–369. 20539944 10.1093/abbs/gmq038

[14] Hodzic J, Giovannetti E, Diosdado B, Adema AD, Peters GJ (2011) Regulation of deoxycytidine kinase expression and sensitivity to gemcitabine by micro-RNA 330 and promoter methylation in cancer cells. Nucleosides Nucleotides Nucleic Acids 30: 1214–1222. 22132977 10.1080/15257770.2011.629271

[15] Giachino C, Lantelme E, Lanzetti L, Saccone S, Bella Valle G, et al (1997) A novel SH3-containing human gene family preferentially expressed in the central nervous system. Genomics 41: 427–434. 9169142 10.1006/geno.1997.4645

[16] Ringstad N, Nemoto Y, De Camilli P (1997) The SH3p4/Sh3p8/SH3p13 protein family: binding partners for synaptojanin and dynamin via a Grb2-like Src homology 3 domain. Proc Natl Acad Sci U S A 94: 8569–8574. 9238017 10.1073/pnas.94.16.8569 PMC23017

[17] Dasgupta S, Jang JS, Shao C, Mukhopadhyay ND, Sokhi UK, et al (2013) SH3GL2 is frequently deleted in non-small cell lung cancer and downregulates tumor growth by modulating EGFR signaling. J Mol Med (Berl) 91: 381–393. 22968441 10.1007/s00109-012-0955-3 PMC3691869

[18] Shang C, Guo Y, Fu S, Fu W, Sun K (2010) SH3GL2 gene participates in MEK-ERK signal pathway partly by regulating EGFR in the laryngeal carcinoma cell line Hep2. Med Sci Monit 16: BR168–173. 20512084

[19] Potter N, Karakoula A, Phipps KP, Harkness W, Hayward R, et al (2008) Genomic deletions correlate with underexpression of novel candidate genes at six loci in pediatric pilocytic astrocytoma. Neoplasia 10: 757–772. 18670637 10.1593/neo.07914 PMC2481566

[20] Soubeyran P, Kowanetz K, Szymkiewicz I, Langdon WY, Dikic I (2002) Cbl-CIN85-endophilin complex mediates ligand-induced downregulation of EGF receptors. Nature 416: 183–187. 11894095 10.1038/416183a

[21] Johnson H, Del Rosario AM, Bryson BD, Schroeder MA, Sarkaria JN, et al (2012) Molecular characterization of EGFR and EGFRvIII signaling networks in human glioblastoma tumor xenografts. Mol Cell Proteomics 11: 1724–1740. 22964225 10.1074/mcp.M112.019984 PMC3518138

[22] Wang SC, Hung MC (2009) Nuclear translocation of the epidermal growth factor receptor family membrane tyrosine kinase receptors. Clin Cancer Res 15: 6484–6489. 19861462 10.1158/1078-0432.CCR-08-2813 PMC5537741

[23] Mangelberger D, Kern D, Loipetzberger A, Eberl M, Aberger F (2012) Cooperative Hedgehog-EGFR signaling. Front Biosci (Landmark Ed) 17: 90–99. 22201734 10.2741/3917 PMC3284771

[24] Brand TM, Iida M, Wheeler DL (2011) Molecular mechanisms of resistance to the EGFR monoclonal antibody cetuximab. Cancer Biol Ther 11: 777–792. 21293176 10.4161/cbt.11.9.15050 PMC3100630

[25] Taylor TE, Furnari FB, Cavenee WK (2012) Targeting EGFR for treatment of glioblastoma: molecular basis to overcome resistance. Curr Cancer Drug Targets 12: 197–209. 22268382 10.2174/156800912799277557 PMC3464093

[26] Hong Y, Sang M, Shang C, Xue YX, Liu YH (2012) Quantitative analysis of topoisomerase II alpha and evaluation of its effects on cell proliferation and apoptosis in glioblastoma cancer stem cells. Neurosci Lett 518: 138–143. 22569122 10.1016/j.neulet.2012.04.071

[27] Zhang FL, Wang P, Liu YH, Liu LB, Liu XB, et al (2013) Topoisomerase I inhibitors, shikonin and topotecan, inhibit growth and induce apoptosis of glioma cells and glioma stem cells. PLoS One 8: e81815. 24303074 10.1371/journal.pone.0081815 PMC3841142

[28] Stiles CD, Rowitch DH (2008) Glioma stem cells: a midterm exam. Neuron 58: 832–846. 18579075 10.1016/j.neuron.2008.05.031

[29] Qu S, Yao Y, Shang C, Xue Y, Ma J, et al (2012) MicroRNA-330 is an oncogenic factor in glioblastoma cells by regulating SH3GL2 gene. PLoS One 7: e46010. 23029364 10.1371/journal.pone.0046010 PMC3448729

[30] Li Y, Zhu X, Xu W, Wang D, Yan J (2013) miR-330 regulates the proliferation of colorectal cancer cells by targeting Cdc42. Biochem Biophys Res Commun 431: 560–565. 23337504 10.1016/j.bbrc.2013.01.016

[31] Mao Y, Chen H, Lin Y, Xu X, Hu Z, et al (2013) microRNA-330 inhibits cell motility by downregulating Sp1 in prostate cancer cells. Oncol Rep 30: 327–333. 23670210 10.3892/or.2013.2452

[32] Zheng ZM, Wang X (2011) Regulation of cellular miRNA expression by human papillomaviruses. Biochim Biophys Acta 1809: 668–677. 21616186 10.1016/j.bbagrm.2011.05.005 PMC3175324

[33] Sinha S, Chunder N, Mukherjee N, Alam N, Roy A, et al (2008) Frequent deletion and methylation in SH3GL2 and CDKN2A loci are associated with early- and late-onset breast carcinoma. Ann Surg Oncol 15: 1070–1080. 18239974 10.1245/s10434-007-9790-0

[34] Majumdar S, Gong EM, Di Vizio D, Dreyfuss J, Degraff DJ, et al (2013) Loss of Sh3gl2/endophilin A1 is a common event in urothelial carcinoma that promotes malignant behavior. Neoplasia 15: 749–760. 23814487 10.1593/neo.121956 PMC3689238

[35] Maiti GP, Mondal P, Mukherjee N, Ghosh A, Ghosh S, et al (2013) Overexpression of EGFR in head and neck squamous cell carcinoma is associated with inactivation of SH3GL2 and CDC25A genes. PLoS One 8: e63440. 23675485 10.1371/journal.pone.0063440 PMC3651136

[36] de Moor CH, Meijer H, Lissenden S (2005) Mechanisms of translational control by the 3′ UTR in development and differentiation. Semin Cell Dev Biol 16: 49–58. 15659339 10.1016/j.semcdb.2004.11.007

[37] Robins H, Press WH (2005) Human microRNAs target a functionally distinct population of genes with AT-rich 3′ UTRs. Proc Natl Acad Sci U S A 102: 15557–15562. 16230613 10.1073/pnas.0507443102 PMC1257391

[38] Henson ES, Gibson SB (2006) Surviving cell death through epidermal growth factor (EGF) signal transduction pathways: implications for cancer therapy. Cell Signal 18: 2089–2097. 16815674 10.1016/j.cellsig.2006.05.015

[39] Kirisits A, Pils D, Krainer M (2007) Epidermal growth factor receptor degradation: an alternative view of oncogenic pathways. Int J Biochem Cell Biol 39: 2173–2182. 17855153 10.1016/j.biocel.2007.07.012

[40] Dhillon AS, Hagan S, Rath O, Kolch W (2007) MAP kinase signalling pathways in cancer. Oncogene 26: 3279–3290. 17496922 10.1038/sj.onc.1210421

[41] Dudu V, Able RA Jr, Rotari V, Kong Q, Vazquez M (2012) Role of Epidermal Growth Factor-Triggered PI3K/Akt Signaling in the Migration of Medulloblastoma-Derived Cells. Cell Mol Bioeng 5: 502–413. 24273611 10.1007/s12195-012-0253-8 PMC3832994

[42] Chen J (2012) Roles of the PI3K/Akt pathway in Epstein-Barr virus-induced cancers and therapeutic implications. World J Virol 1: 154–161. 24175221 10.5501/wjv.v1.i6.154 PMC3782276

[43] Ramjaun AR, Angers A, Legendre-Guillemin V, Tong XK, McPherson PS (2001) Endophilin regulates JNK activation through its interaction with the germinal center kinase-like kinase. J Biol Chem 276: 28913–28919. 11384986 10.1074/jbc.M103198200

[44] Lin A, Dibling B (2002) The true face of JNK activation in apoptosis. Aging Cell 1: 112–116. 12882340 10.1046/j.1474-9728.2002.00014.x

[45] Vucic D, Dixit VM, Wertz IE (2011) Ubiquitylation in apoptosis: a post-translational modification at the edge of life and death. Nat Rev Mol Cell Biol 12: 439–452. 21697901 10.1038/nrm3143

[46] Lin H, Chen C, Li X, Chen BD (2002) Activation of the MEK/MAPK pathway is involved in bryostatin1-induced monocytic differenciation and up-regulation of X-linked inhibitor of apoptosis protein. Exp Cell Res 272: 192–198. 11777344 10.1006/excr.2001.5417

[47] Nesterov A, Lu X, Johnson M, Miller GJ, Ivashchenko Y, et al (2001) Elevated AKT activity protects the prostate cancer cell line LNCaP from TRAIL-induced apoptosis. J Biol Chem 276: 10767–10774. 11278284 10.1074/jbc.M005196200

[48] Gibson EM, Henson ES, Haney N, Villanueva J, Gibson SB (2002) Epidermal growth factor protects epithelial-derived cells from tumor necrosis factor-related apoptosis-inducing ligand-induced apoptosis by inhibiting cytochrome c release. Cancer Res 62: 488–496. 11809700

[49] Henson ES, Gibson EM, Villanueva J, Bristow NA, Haney N, et al (2003) Increased expression of Mcl-1 is responsible for the blockage of TRAIL-induced apoptosis mediated by EGF/ErbB1 signaling pathway. J Cell Biochem 89: 1177–1192. 12898516 10.1002/jcb.10597

[50] Johnstone RW, Frew AJ, Smyth MJ (2008) The TRAIL apoptotic pathway in cancer onset, progression and therapy. Nat Rev Cancer 8: 782–798. 18813321 10.1038/nrc2465

[51] Salvesen GS, Duckett CS (2002) IAP proteins: blocking the road to death’s door. Nat Rev Mol Cell Biol 3: 401–410. 12042762 10.1038/nrm830

[52] Fulda S (2009) Cell death in hematological tumors. Apoptosis 14: 409–423. 19130230 10.1007/s10495-008-0306-6

[53] Jun T, Gjoerup O, Roberts TM (1999) Tangled webs: evidence of cross-talk between c-Raf-1 and Akt. Sci STKE 1999: PE1. 10.1126/stke.1999.13.pe1 11865188

[54] Aksamitiene E, Kiyatkin A, Kholodenko BN (2012) Cross-talk between mitogenic Ras/MAPK and survival PI3K/Akt pathways: a fine balance. Biochem Soc Trans 40: 139–146. 22260680 10.1042/BST20110609

[55] Chandarlapaty S (2012) Negative feedback and adaptive resistance to the targeted therapy of cancer. Cancer Discov 2: 311–319. 22576208 10.1158/2159-8290.CD-12-0018 PMC3351275

